# Supplementary material for: Wellbeing Impact Study of High-Speed 2 (WISH2): Protocol for a mixed-methods examination of the impact of major transport infrastructure development on mental health and wellbeing
Source: PLoS One. 2024 Feb 29;19(2):e0298701. doi: 10.1371/journal.pone.0298701 (PMC10903902; doi:10.1371/journal.pone.0298701)
Supplement: S1 File — (PDF) [file pone.0298701.s001.pdf]

WISH2-WP3-W1-D6

WISH2 qualitative topic guide

Local resident's topic guide for focus groups

- My name is [name] and I am [role] at RAND Europe. I am here with my colleague [name] who will be supporting and taking notes.
    - RAND Europe is a not-for-profit policy research organisation.
- We are working with the University of Cambridge to conduct an independent study examining the impact of HS2 on mental health and wellbeing.
    - This study is funded by the Department for Transport and High-Speed Two Ltd, supported by the National Institute for Health Research. The study will be monitored in line with the National Institute for Health Research (NIHR) standard contracting and monitoring processes to ensure independence and objectivity.
- The purpose of this discussion today is to better understand positive and negative effects of the planning and activities to date, as well as the cancellation of HS2, on local residents' mental health and wellbeing.
- The discussion will take around 60 minutes if online or 60-120 in-person.
- *Facilitator to ask about and explain the following aspects:*
    - We have shared with you the information sheet and consent forms which outlined what we're doing, why and what we will do with data. Since we have not received the signed consent forms from all, we would like to record taking your verbal consent. We hope this is OK **Turn on audio recorder (if OK).**
    - This is just to remind us all that, as per the information sheet and consent forms:
        - Taking part today is voluntary: you can stop or leave at any point, without giving a reason.
        - We'll be recording the session so that we don't have to scribble everything down.
        - The recording will be sent to a secure transcription service to provide a transcript. The transcript will be anonymised which means the transcriber we will take out any text that could identify you, such as your name or where you live. We will publish the findings of our research, but we will not identify you personally in any publications.
        - We won't be sharing any of your personal details (like your name, address or email address) with anyone outside of the research team.
          We understand that your participation here today confirms that you are happy with this and that we can begin.
- *Facilitator to explain housekeeping rules for the focus group:*
    - I will be leading the discussion and my colleague will be here to provide support, take notes, and check if everyone in the group is doing okay
    - Chatham House rule: participants are free to use the information they receive during the discussion, but they are not allowed to reveal the identity or affiliation of the speaker(s), nor that of any other participant.
    - All views are welcome and please do feel comfortable chipping-in and sharing thoughts as they arise, but we should also try to avoid speaking over each other as much as possible. Feel free to raise your hand and we will come to you.
    - If you feel comfortable to share these in a group forum, we would welcome your personal perspectives and experiences. We would also be interested to hear about experiences of close friends, families or neighbours.
- Do you have any questions before we start?

- Let's begin by going round the group and introducing ourselves.

1. Recognising that there is no right or wrong answer here, how much (if anything) do you know about the plans for, then subsequent cancellation of HS2 where you live?
    a. Is this something you keep a close eye on (and why/why not)?
    b. *If participant knows about HS2 locally:* What type of HS2 construction (if any) was planned near you?
    c. What did you think about the information available regarding the timelines for HS2 construction near where you live (and why)? Prompt: How did you hear about the cancellation? Did/do you have enough information, how accessible was it, did you know where to find it, is it clear?
    d. Would you say you are more in favour of the HS2 project, or against it?
        i. *If against:* Have you taken part in any direct action/protests against the HS2 project? For example, have you taken part in a protest or written to your MP/counsellor/mayor/local press?
        ii. *If for:* Have you taken part in any direct action in support of the HS2 project? For example, have you taken part in any demonstrations, attending meetings or written to your MP/counsellor/mayor/local press?
        iii. *All:* How did you feel about the cancellation and why?

2. Do you think HS2 (planning and cancellation) has had an impact on your own mental health and wellbeing? Why?
   Are these related to planning or cancellation of HS2?
   Ask the open question and then ask each of the following in turn if not already covered by participants.
   *Explore **how** these impacts occur and **how they might be linked to mental health/wellbeing***
   Prompts for facilitator - impacts could be related to:
    a. Employment and local business in your local area
    b. Your local environment (such as green spaces, biodiversity, visual appeal, noise/air pollution)
    c. Your local community (such as how connected you feel to your community and existing networks and social circles)
    d. Travelling around your local area (for commuting and other travel, e.g. traffic, cost of transport)
    e. Your financial situation
    f. Housing in your local area (such as house prices, (in)voluntary re-location), e.g., did you move house as a result of HS2? Was your land/area around your house directly impacted by HS2?
    g. Access to local services (e.g. healthcare, social care/support, sports/leisure facilities, cultural facilities)

3. *For any negative impacts described:* Are you adapting to, or managing with the impacts on your mental health and wellbeing?
    a. *If not*: Why?
    b. *If yes:* How are you managing or dealing with the impacts?
    c. Is there anything you think may be making it easier or more difficult to deal with the impacts?

      i. *If yes*: what? Have you connected with anyone from your neighbourhood/community on this issue (informal chats, organised groups, etc) Have you engaged with the HS2 Help Desk or the local HS2 community engagement teams?

   d. What, if any, additional resources or support do you need to help deal with these impacts (e.g. social support, financial support, healthcare support)?

      i. *Interviewer to note that it is not possible for the research team to provide any additional resources but analysis of the resource needs highlighted by residents will be shared with HS2 Ltd.*

4. Thinking about the future do you think you'll still be seeing the impacts you described earlier, or do you think they will have changed or no longer be present?
   a. Why?
   b. What other or additional impacts might you expect to see as a result of HS2 in the future?
   c. *Wave 2 and 3 participants only:* Are the impacts of the HS2 change to the route in your area the same as you expected to see when the changes were first announced?
      i. *If not:* How were these impacts different to what you initially expected?

5. [question(s) exploring some of the key themes arising from previous work packages - TBC.]

6. Is there anything else you would like to add that we haven't covered already?

- *Facilitator to round the discussion off, including:*
  - *A summary of what has been discussed*
  - *An explanation of what will happen next (transcription of session, analysis, etc.) and the next point of contact*
  - *Thanking participants for their contributions*
  - *Signposting to Samaritans for support with mental health (tel. 116 123 - put in chat or share verbally if in person)*
