# Supplementary material for: Wellbeing Impact Study of High-Speed 2 (WISH2): Protocol for a mixed-methods examination of the impact of major transport infrastructure development on mental health and wellbeing
Source: PLoS One. 2024 Feb 29;19(2):e0298701. doi: 10.1371/journal.pone.0298701 (PMC10903902; doi:10.1371/journal.pone.0298701)
Supplement: S2 File — (PDF) [file pone.0298701.s002.pdf]

WISH2-WP3-W1-D5

WISH2 qualitative topic guide

Local resident's topic guide for interviews (changed route)

- My name is [name] and I am [role] at RAND Europe.
    - RAND Europe is a not-for-profit policy research organisation.
- We are working with the University of Cambridge to conduct an independent study examining impact of HS2 on mental health and wellbeing.
    - This study is funded by the Department for Transport and High-Speed Two Ltd, supported by the National Institute for Health Research. These organisations will not try to influence our findings.
- The purpose of this interview today is to better understand positive and negative effects of the planning and cancellation of HS2 on residents' mental health and wellbeing.
- The interview will take around 30-45 minutes. Your participation is optional, and you can withdraw from the interview at any point.
- *Interviewer to ask about and explain the following aspects:*
    - Thanks for returning the consent form. You have said you're happy for me to record our discussion today so I will start the recording now if you are happy with that still? ***Turn on audio recorder (if OK).***
    - Can you please confirm that you have read the information about the study we shared with you (including the participant information sheet). *If not, summarise info:*
        - Taking part today is voluntary, you do not have to take part and you don't have to answer anything you don't want to. During our session, you can stop or leave at any point, without giving me a reason.
        - We'll be taking notes but recording means that we don't have to scribble everything down.
        - The recording of the conversation will be sent to a secure transcription service. They will listen to the recording and write out the conversation as a transcript. The transcript will be anonymised by the research team, which means we will take out any text that could identify you, such as your name or where you live. We will publish the findings of our research, but we will not identify you personally in any publications.
        - We won't be sharing any of your personal details (like your name, address or email address) with anyone outside of the research team.
- Do you have any questions before we start?

1. Could you tell me a little bit about yourself?
    a. Could you tell me a bit about your household (e.g., who do you live with, how old are you, occupation(s),
    b. How long have you lived in your neighbourhood, and how settled/connected do you feel to you neighbourhood/ local area? *Prompt:* how do you feel about the opportunities for socialising in your area?

2. Recognising that there is no right or wrong answer here, how much (if anything) do you know about the plans for, and then subsequent cancellation of HS2 where you live?

Version 2-0. 09/11/2022. IRAS ID: 302856

   a. Is this something you kept/keep a close eye on (and why/why not)?
   b. *If interviewee knows about HS2 locally:* What type of HS2 construction was planned near you?
   c. What do you think about the information available regarding the cancellation of the route of HS2near where you live (and why)? *Prompt:* How did you hear about the change to the route? Do you have enough information, how accessible is it, do you know where to find it, is it clear?
   d. Would you say you are more in favour of the HS2 project, or against it?
      i. *If against:* Have you taken part, or are considering taking part, in any direct action/protests against the HS2 project? For example, have you taken part in a protest or written to your MP/counsellor/mayor/local press?
      ii. *If for:* Have you taken part, or are considering taking part, in any direct action in support of the HS2 project? For example, have you taken part in any demonstrations, attending meetings or written to your MP/counsellor/mayor/local press?
      iii. *All:* How did you feel about the cancellation of HS2 construction near where you live and why?

3. How would you describe your overall health and wellbeing?
4. Do you think HS2 (planning and cancellation) will have, or is already having, an impact on your own mental health and wellbeing? *If yes:* will it be, or is it already, positive or negative impact? Why?
   *Ask the open question and then ask each of the following in turn if not already covered by respondent. Explore **<u>how</u>** these impacts occur and **<u>how they might be linked to mental health/wellbeing</u>***
   Prompt: impacts could be related to:
   a. Employment and local business in your local area
   b. Your local environment (such as green spaces, biodiversity, visual appeal, noise/air pollution)
   c. Your local community (such as how connected you feel to your community and existing networks and social circles)
   d. Travelling around your local area (for commuting and other travel, e.g. traffic, cost of transport)
   e. Your financial situation
   f. Housing in your local area (such as house prices, (in)voluntary re-location)
   g. Access to local services (e.g. healthcare, social care/support, sports/leisure facilities, cultural facilities)
   h. Your home/living situation
      i. *If yes,* can you please explain how? E.g., were you forced to sell your house? Was your land/area around your house directly impacted by HS2? Did you choose to sell your home after hearing of the HS2 plans in your area? Did you ask the government to purchase your property after hearing plans? (Service a Blight Notice)? Was your house devalued by the implementation of the line?
      ii. *If clarification needed*: Can you give us a brief description of timelines, in terms of finding out about HS2, moving home (if relevant), hearing about the change to the route, etc.

5. If there is one, what has been the most significant change for you or for anyone else from your household (or your community) that has resulted from HS2? *Interviewer to categorise the change (see a-f below); If no story/experience for them personally, do they know about anyone else's story*
    a. What was the most significant change for you/them?
    b. When did it happen and to whom?
    c. Where did it happen?
    d. Why is this the most significant or important for you/them?
    e. How have you/they managed or adapted?
    f. What would the title of this story be?

6. *For any negative impacts described:* Are you adapting to or managing with the impacts you just described?
    a. *If not*: Why?
    b. *If yes:* How are you adapting to or managing/dealing with the impacts?
    c. Is there anything you think may be making it easier or more difficult to deal with the impacts?
        i. *If yes*: what? E.g., Have you connected with anyone from your neighbourhood/community on this issue (informal chats, organised groups, etc). Have you engaged with the HS2 Help Desk or the local HS2 community engagement teams?
    d. What, if any, additional resources or support do you need to help deal with these impacts (e.g. social support, financial support, healthcare support)?
        i. *Interviewer to note that it is not possible for the research team to provide any additional resources but analysis of the resource needs highlighted by residents will be shared with HS2 Ltd.*
    e. Did you receive any financial support from HS2?
        i. *If yes:* what kind of financial support did you receive? how easy or difficult was the process of receiving it (and how could it be improved)? how fair or adequate do you find the financial support you received at the end (why)?
        ii. *If not:* why not? were you aware of the available financial support? were you eligible (why not)?

7. Thinking about the future (immediate future or further in the future), do you think you'll still be seeing/experiencing the impacts you described earlier, or do you think they will have changed or no longer be present?
    a. Why?
    b. What other or additional impacts might you expect to see as a result of HS2 (and the cancellation) in the future?
    c. *Wave 2 and 3 participants only:* Are the impacts of the HS2 change to the route in your area the same as those you expected to see when the changes were announced?
        i. *If not:* How are these impacts different to what you initially expected?

8. Do you think there are any particular groups of people who may feel the impacts you described more (either positively or negatively), e.g. low-income households, women, ethnic minorities, children, disabled/frail, older age? Why? And how in particular may they be impacted?

9. [question(s) exploring some of the key themes arising from previous work packages - TBC.]

10. Is there anything else you would like to add that we haven't covered already?
