# Supplementary material for: Wellbeing Impact Study of High-Speed 2 (WISH2): Protocol for a mixed-methods examination of the impact of major transport infrastructure development on mental health and wellbeing
Source: PLoS One. 2024 Feb 29;19(2):e0298701. doi: 10.1371/journal.pone.0298701 (PMC10903902; doi:10.1371/journal.pone.0298701)
Supplement: S3 File — (PDF) [file pone.0298701.s003.pdf]

WISH2-WP3-W1-D9

WISH2 qualitative topic guide

Local stakeholder's topic guide for interviews (changed route)

- My name is [name] and I am [role] at RAND Europe.
    - RAND Europe is a not-for-profit policy research organisation.
- We are working with the University of Cambridge to conduct an independent study examining impact of HS2 on mental health and wellbeing.
    - This study is funded by the Department for Transport and High-Speed Two Ltd, supported by the National Institute for Health Research. These organisations will not try to influence our findings.

- As you know, we are conducting a study examining impact of HS2 on mental health and wellbeing (or understand where there is no impact).
- The purpose of this interview today is to better understand positive and negative effects of the planning and change/cancellation to HS2 on local residents and stakeholders' mental health and wellbeing.

- The interview will take around 30-45 minutes. Your participation is optional, and you can withdraw from the interview at any point.
  Interviewer to ask about and explain the following aspects:
    - Thanks for returning the consent form. You have said you're happy for me to record our discussion today so I will start the recording now if you are happy with that still? ***Turn on audio recorder (if OK)***.
    - Can you please confirm that:
        - You read the information about the study we shared with you (including the participant information sheet). *If not, summarise info:*
            - Voluntary. You don't have to take part and don't have to answer anything you don't want to - free to withdraw from study at any time.
            - Anonymous. Your personal details will not be disclosed to third parties at any stage of the process. Data from this interview will be analysed and may be used in the final report that may be published. However, the analysis will be written up in such a way that it will not be feasible to identify any individual.
            - Recorded: We'll be taking notes but recording means that we don't have to scribble everything down.
    - Do you have any questions before we start?

1. Could you tell me a little bit about yourself, your role and the organisation you work for? *If an activist/voluntary community organisation probe on impact of belonging to such a group or organisation if relevant (but be cautious to keep conversation at a community rather than personal level)*

2. How much (if anything) do you know about the plans for, then subsequent change/cancellation of HS2 your community/organisation (or generally if taking a broader/national perspective)?
    a. Is this something you keep a close eye on (and why/why not)?
    b. *If interviewee knows about HS2 locally:* What type of HS2 construction was planned near your community/organisation?

c. How did you hear about the change/cancellation?
d. What do you think about the information available regarding the change/cancellation of HS2 near your community/organisation (and why)?
   Prompt: do you have enough information, how accessible is it, do you know where to find it, is it clear?
e. Would you say your community/organisation have been more in favour of the HS2 project, or against it?
   a. *If against:* Have you taken part in any direct action/ protests against the HS2 project? For example, have you taken part in a protest or written to your MP/counsellor/mayor/local press?
   b. *If for:* Have you taken part in any direct action in support of the HS2 project? For example, have you taken part in any demonstrations, attending meetings or written to your MP/counsellor/mayor/local press?
f. If possible, could you speak to the feeling within your organisation/community about the change/cancellation? (and why)?

3. Do you think HS2 (planning and change to the route/cancellation) will have, or is already having, an impact on your community's mental health and wellbeing? *If yes:* Will it be, or is it already, a positive or negative impact? Why?
   *Encourage the respondent to share stories (if they have any) on how HS2 has led to the impacts they described. Ask the open question and then ask each of the following in turn if not already covered by respondent. Explore **how** these impacts occur and **how they might be linked to mental health/wellbeing***
   Prompt: impacts could be related to:
   a. Employment and local business in the local area
   b. The local environment (such as green spaces, biodiversity, visual appeal, noise/air pollution)
   c. The local community (such as how connected the community is and existing networks and social circles)
   d. Travelling around the local area (for commuting and other travel, e.g. traffic, cost of transport)
   e. Financial situations
   f. Housing in the local area (such as house prices, (in)voluntary re-location). *Prompts:* residents being forces to sell their house; land/area near residents being directly impacted by HS2; residents choosing to sell their homes/requesting that it bought by HS2
   g. Access to local services (e.g. healthcare, social care/support, sports/leisure facilities, cultural facilities)
   h. Other

4. We're interested in stories as these can be a good way of understanding and communicating experiences. With this in in mind, of the impact(s) just described, which caused the most significant change for your community?

5. *Interviewer to categorise the change (see a-f below); If no story/experience for their community/organisation, do they know about any other communities/organisations' story?*
    a. When did it happen and to whom?
    b. Where did it happen?
    c. Why is this the most significant or important for the community?
    d. What would the title of this story be?
    e. Were there any examples of adapting/managing these impacts? *(then frame the next question about adaptation as relating to the community as a whole)*

6. *For any negative impacts described:* How have you seen the community adapting to or managing the impacts you just described?
    a. Is there anything you think may be facilitating or preventing the community from adapting to/managing the impacts you described?
    b. Does the community need any additional resources and support to help deal with these impacts (e.g., social support, financial support, healthcare support)?
        a. Have residents mentioned using an HS2 Help Desk or local HS2 community engagement team?
        b. *Interviewer to note that it is not possible for the research team to provide any additional resources, but analysis of the resource needs highlighted by residents will be shared with HS2 Ltd.*

7. Do you think there are any particular groups of people who feel the impacts you described more (either positively or negatively), e.g. low-income households, women, ethnic minorities, children, disabled/frail, older age?
    a. Why? And how in particular are they impacted?

8. Thinking about the future (immediate future or further in the future, now that the line has been changed/cancelled), do you think the impacts you described earlier will still be present or do you think they will have changed or no longer be present?
    a. Why?
    b. What other or additional impacts might you expect to see as a result of HS2 (and the change to the route next to you) in the future?
    c. *Wave 2 and 3 participants only:* Are the impacts of the change to the route in your area the same as those you expected to see when the changes were announced?
        i. *If not:* How are these impacts different to what you initially expected?

9. [question(s) exploring some of the key themes arising from previous work packages - TBC.]

10. Is there anything else you would like to add that we haven't covered already?
